# Rapid Fabrication of Smooth Micro-Optical Components on Glass by Etching-Assisted Femtosecond Laser Modification

**DOI:** 10.3390/ma15020678

**Published:** 2022-01-17

**Authors:** Bao-Xu Wang, Jin-Yong Qi, Yi-Ming Lu, Jia-Xin Zheng, Ying Xu, Xue-Qing Liu

**Affiliations:** State Key Laboratory of Integrated Optoelectronics, College of Electronic Science and Engineering, Jilin University, Changchun 130012, China; bxwang_sklio@jlu.edu.cn (B.-X.W.); q1j2y33@163.com (J.-Y.Q.); 13180893370@163.com (Y.-M.L.); zhengjx19@163.com (J.-X.Z.); xuying1969@hotmail.com (Y.X.)

**Keywords:** foturan glass microlens, laser direct writing, profile scanning, wet etching

## Abstract

Femtosecond laser (fs-laser) is unfavorable in applications for the fabrication of micro-optical devices on hard materials owing to the problems of low fabrication efficiency and high surface roughness. Herein, a hybrid method combining fs-laser scanning, subsequent etching, and annealing was proposed to realize micro-optical devices with low roughness on glass. Compared to traditional laser ablation, the fabrication efficiency in this work was improved by one order of magnitude, and the surface roughness was decreased to 15 nm. Using this method, aspherical convex microlenses and spherical concave microlenses that possess excellent focusing and imaging properties are realized on photosensitive glass. The diameter and height of the microlenses were controlled by adjusting the fabrication parameters. These results indicate that the fs-laser-based hybrid method will open new opportunities for fabricating micro-optical components on hard materials.

## 1. Introduction

With the miniaturization and integration of modern optical systems, micro-optical components exhibit notable potential applications in virtual and augmented reality [1,2,3], autonomous navigation [4], medical monitoring [5,6], and laser shaping [7,8]. Among these components, microlenses and their arrays have been widely investigated and rapidly developed owing to their easy integration and broad functions [9,10,11,12,13]. A large number of technologies have been proposed to fabricate microlenses, such as photolithography [14], electron beam lithography [15], nanoimprint [16], and etching and fs-laser direct writing (FsLDW) [17,18]. For example, Yu et al. used electrohydrodynamic jet printing to fabricate a layered microlens array on a polydimethylsiloxane film and then used a microfluidic chip to deform the microlens array film to create an artificial compound eye with a variable field of view, ranging from 0° to 160° [19]. Jeong et al. reported a new microfabrication method for a multifocus microlens array using multilayer lithography and thermal reflow to extend the depth of field [20]. To date, most of these methods cannot fabricate microlenses with designable and complex profiles. Various FsLDW-based technologies exhibit a stupendous capacity to process arbitrary complex three-dimensional (3D) micro/nanostructures, including femtosecond laser multiphoton polymerization, femtosecond laser ablation, etching-assisted femtosecond laser modification, etc., have already been used to fabricate polymer micro-optical components with good profiles [21,22,23,24,25]. To enhance the stability and broaden the optical transmission region, it is necessary to fabricate micro-optical components on hard materials. When fs-laser ablation is used to process hard materials, laser-induced micro/nanostructures and scattered particles are formed on the surface, which has a serious impact on the performance of the micro-optical components [26,27,28,29]. Although laser polishing has been proposed to improve the surface quality [30,31], it does not satisfy the high requirement for applications in optical devices.

Recently, etching-assisted fs-laser modification has been proposed to fabricate micro-optical components on hard materials with high smoothness [32,33,34]. For example, Chen et al. successfully fabricated microlens arrays (surface roughness of 56 nm) on silica glass by wet etching-assisted fs-laser modification [35]. However, this method can only fabricate concave microlenses with spherical profiles.

In this study, a hybrid method combined with femtosecond laser scanning, subsequent etching, and thermal annealing was proposed to fabricate smooth micro-optical components on glass. Compared with direct laser ablation, the fabrication efficiency and surface quality of the micro-optical components can be significantly improved by the hybrid methods. To demonstrate its potential, we fabricated and characterized various convex and concave microlens with good 3D morphology and high surface smoothness.

## 2. Materials and Methods

**Figure 1:**
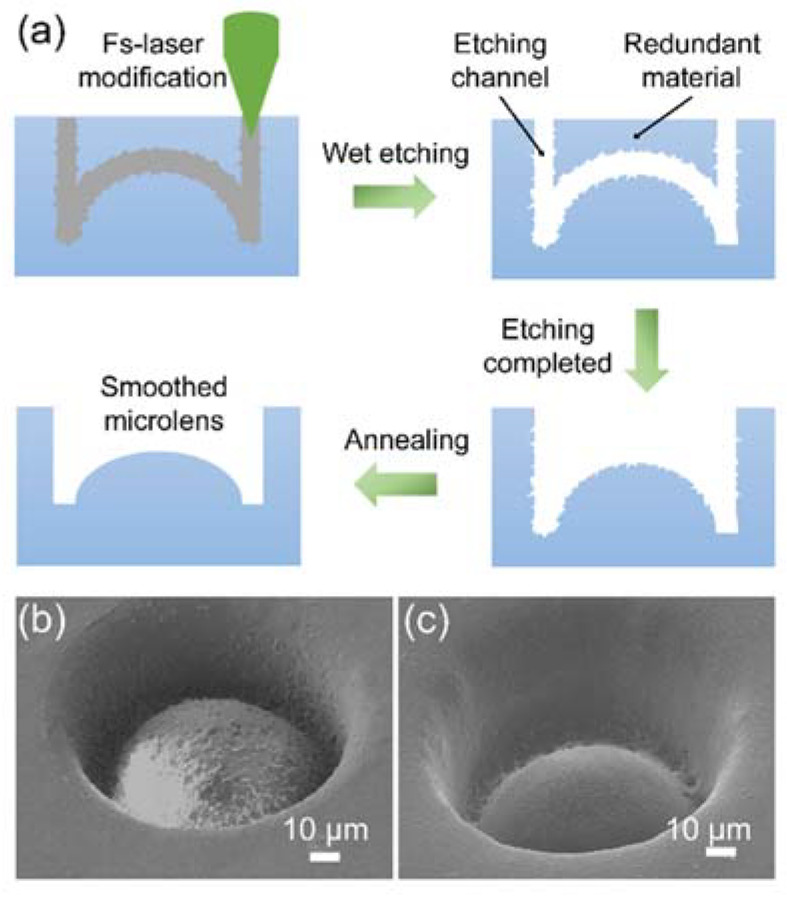
(**a**) Diagram of the mixing process. (**b**,**c**) SEM images of the microlens before and after the second annealing.

As a proof-of-concept, we fabricated a convex microlens on a commercial photosensitive glass (Foturan, Schott, Mainz, Germany). A schematic of the hybrid fabrication method is presented in Figure 1a. First, a modified region was formed by a focused second harmonic fs-laser beam (515 nm), which was generated by a Pharos fs-laser (Light Conversion). The repetition frequency of the pulsed laser was 200 k; the energy of a single pulse was 20 nJ; the pulse width was 260 fs; the laser was focused by an objective lens (50×, NA = 0.7), and the effective fluence of a single pulse was approximately 70.1 mJ/cm^2^. The scanning was done line by line in a circularly trajectory by moving of a 3D platform. After laser irradiation, the photosensitive glass was processed with the first annealing to crystallize the lithium metasilicate by a tube furnace [36]. The temperature control process of this annealing can be roughly divided into two stages, firstly to 500 °C at a rate of 5 °C min^−1^ and maintained for one hour and secondly to 605 °C at a rate of 3 °C min^−1^ and maintained for one hour. The crystallized region can be facially etched using a hydrofluoric acid (HF) solution with a concentration of 10% in 5 min. Here, a profile scanning strategy was adopted to improve the fabrication efficiency. As shown in Figure 1a, although the redundant materials were not irradiated, they could be removed as a whole block. When the exposure point spacing was 200 nm, assuming a spherical microlens with a radius of 40 μm and depth of 30 μm, the number of laser-irradiated points by scanning of the whole structure (7.7 million) was one order of magnitude larger than that fabricated by the profile scanning method (0.38 million). After the first annealing and wet etching, scanning electron microscopy (SEM) image showed the microlens with poor surface quality (Figure 1b). To improve the surface quality, a second annealing process was performed through heating for 1 h with temperature of 645 °C. Figure 1c shows the SEM image of the fabricated microlens after the second annealing. Compared with the microlens in Figure 1b, the roughness was reduced from 81 nm before annealing to 15 nm after annealing, and the surface quality was significantly improved.

## 3. Results and Discussion

**Figure 2:**
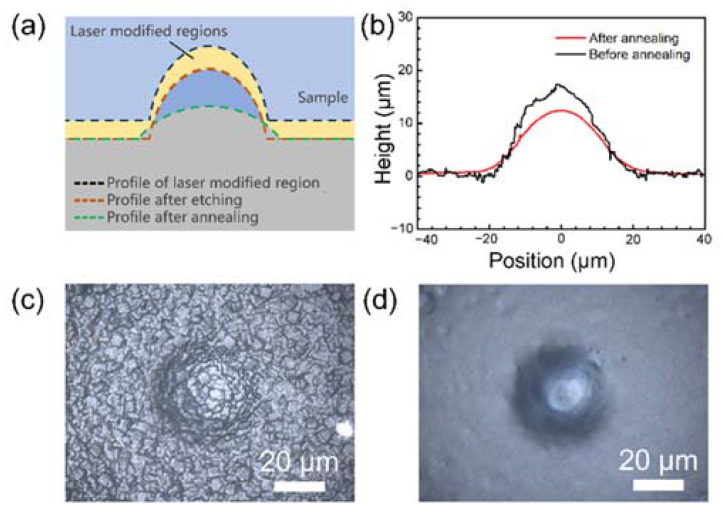
Evolution of microlens morphology during preparation. (**a**,**b**) Schematic diagram and experimental results. (**c**,**d**) Comparison of surface morphology before and after secondary annealing.

Figure 2a shows a schematic diagram of the evolution of the microlens morphology during the manufacturing process. Despite the energy of the laser beam being Gaussian, the lines scanned by the laser have a certain width. Moreover, the width of the line is related to the processing parameters of the laser, including laser energy and scanning point spacing, single-point exposure time, and so on. In the first annealing process, there was no material removal, and the scope of the modified area did not change, except that crystal precipitation occurred in the modified area. During the corrosion removal process, the locations where crystallization occurred in the modified area were removed. Therefore, in this experiment, the overall size of the structure after corrosion was reduced relative to the size of the laser-modified structure, and the amount of reduction depended on the width of the laser-modified line. In the second annealing process, owing to the higher temperature, the glass softened to a certain extent, and the structure tended to be smooth owing to the effect of surface tension. The height of the structure decreased, and the lateral size of the structure increased (as shown in Figure 2b). Moreover, the surface morphology of the structure fluctuated significantly. We used laser scanning confocal microscopy (LSCM, LECT OLS4100, Olympus, Japan) to measure the three-dimensional (3D) surface profile. After annealing, the surface profile of the cross section of the structure was notably improved, as was the smoothness. It can also be seen from the optical photographs in Figure 2c,d that the surface quality of the structure before and after the secondary annealing changed significantly, and the surface smoothness was significantly improved.

**Figure 3:**
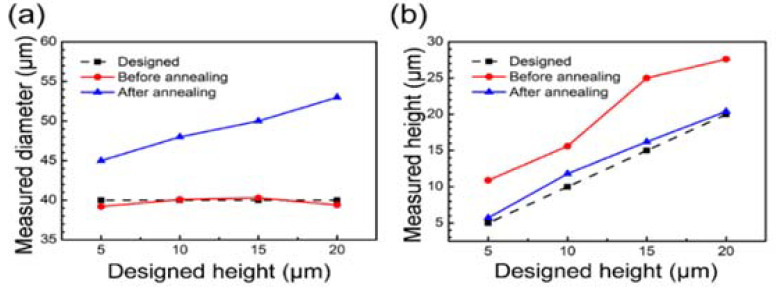
Variation in height and diameter of different microlenses (**a**) before and (**b**) after secondary annealing and the difference with design parameters.

To illustrate the evolution of the morphology of the prepared microlenses with the process, we tested the variation in the height and diameter of the microlenses before and after annealing and the difference with the design parameters. Figure 3a shows that the diameter of the microlens before secondary annealing remained almost the same as the designed value with the designed microlens diameter unchanged. After the secondary annealing, the diameter of the microlens increased with an increase in the designed height. Figure 3b shows the variation in the microlens height. Before secondary annealing, the height of the microlens increased compared to the designed height in all cases. This is because the refractive index mismatch within the dielectric material caused the laser focus to stretch in the propagation direction, and the stretch length varied at different depths from the surface. This resulted in a difference between the height of the structure after laser processing and the designed value. After secondary annealing, the height of the microlens decreased, thus reducing the difference with the designed height. During the annealing process, the surface tension tended to smooth the structure, and the total amount of material did not change before and after annealing, and the reduction in height and the increase in diameter maintained the total amount of material in balance. In conclusion, the above study shows that the diameter and height of the final formed microlens can be controlled by the initial compensation design.

**Figure 4:**
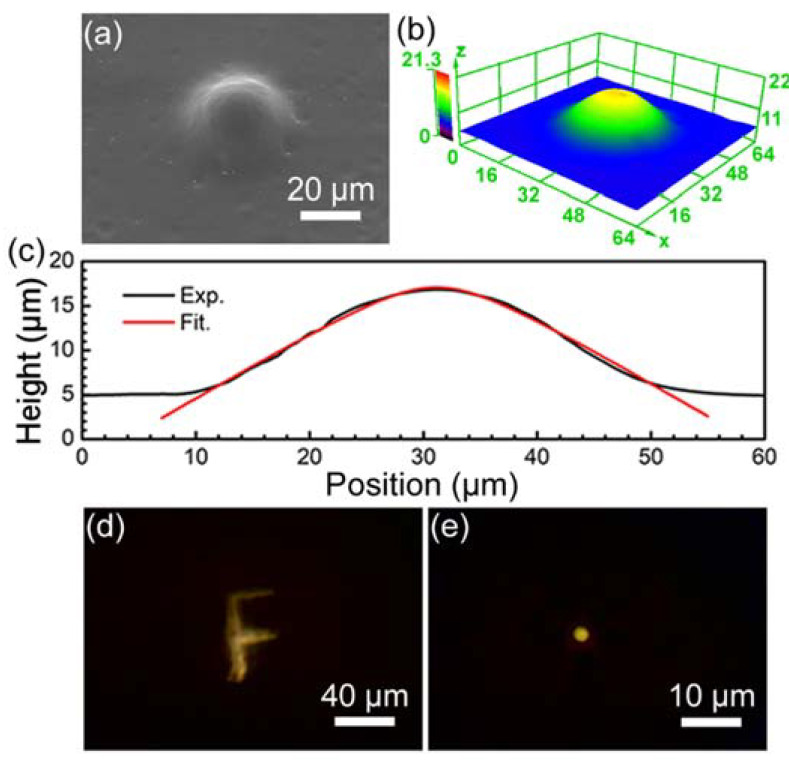
Designed and prepared convex microlenses with diameter and height of 40 μm and 10 μm. (**a**–**c**) are their SEM image, 3D morphology and section profile, respectively. (**d**,**e**) are imaging and focusing performance demonstrations.

Using this method, we designed and successfully fabricated a convex microlens with a diameter of 40 μm and a height of 10 μm. The microlens exhibited good surface morphology and smoothness after wet etching and secondary annealing, as shown in the SEM image and the three-dimensional topography shown in Figure 4a,b, respectively. The black line in Figure 4c shows the cross-sectional profile of this convex microlens, which also demonstrates the excellent surface quality of this convex microlens. According to the principle of geometric optics, with the F-axis as the optical axis of the system, the profile of an axisymmetric aspheric lens in a right-angle coordinate system can be expressed by the following formula:

(1) $$F\left(x\right)=\frac{{\mathrm{cx}}^{2}}{1+\sqrt{1-\left(1+k\right)c^{2}x^{2}}}$$

where x is the ordinate value at each point of the system, c is the curvature at the vertex of the microlens, and k is the conic coefficient of the aspheric surface. According to the different values of k, the aspheric surface shape can be divided into different types, including hyperbolic, parabolic, elliptical, and circular. Here, the red line in Figure 4c shows the fitted profile of the convex microlens in Figure 4a, which is consistent with the experimental data. According to the fitting results, the curvature at the vertex of the microlens (c) is approximately 0.1452, and the conic coefficient of the aspheric surface (k) is approximately −2.76. When the value of k is less than −1, the cross-section profile of the prepared convex microlens is hyperbolic. The optical properties of our prepared convex microlens were also tested. As shown in Figure 4d,e and the results, the convex microlens with aspherical morphology can not only focus light well but also has good imaging capability, which has considerable potential applications in microendoscopy [37], laser beam shaping [38], bioinspired compound eyes [39], and spherical aberration compensation [40].

**Figure 5:**
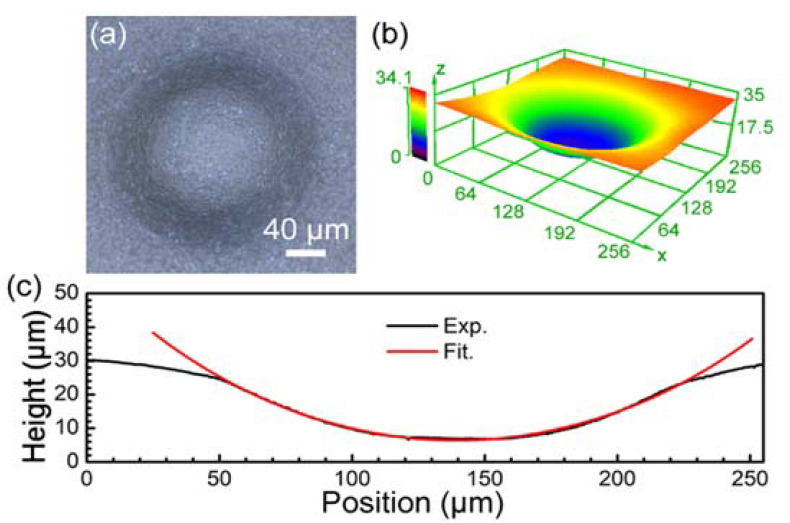
(**a**) LSCM image of concave microlens, (**b**) three-dimensional morphology, and (**c**) cross-sectional profile.

In addition, our proposed method of wet etching-assisted FsLDW can be used to prepare convex microlenses with high surface quality and high surface quality concave microlenses. As shown in the LSCM image in Figure 5a, a concave microlens with high surface smoothness was fabricated on glass. Figure 5b and c show the three-dimensional morphology and cross-sectional profile, respectively. The red line in Figure 5c exhibits the fitted line of the cross-section profile of the concave microlens in Figure 5a. The fitted result indicates that the concave microlens has a spherical profile with a radius of curvature of 220 μm. According to the principle of geometrical optics, the radius of curvature R and focal length f of the spherical microlens satisfy the following equations:

(2) $$R=\frac{h^{2}+{\left(\frac{D}{2}\right)}^{2}}{2h}$$

(3) $$f=\frac{\mathrm{nR}}{\;n\prime -n}$$

where h and D are the height and diameter of the spherical microlens structure prepared in the experiment, respectively, and n and n′ are the refractive indices of air (n = 1) and the material (n′ = 1.515), respectively. The variables h and D are known to be 20 μm and 180 μm, respectively; the radius of curvature R is 212.5 μm, and f is 412.6 μm, where the radius of curvature R and radius obtained by fitting are in good agreement.

## 4. Conclusions

For the foturan photosensitive glass material, we successfully fabricated convex and concave microlenses with good surface quality by etching assisted fs-laser modification. Compared with laser scanning of the entire structure, the processing efficiency can be improved by approximately one order of magnitude using a profile scanning method. In addition, the surface morphology (including the diameter and height) of the microlens can be adjusted by changing the parameters of the subsequent wet etching and annealing treatments. This hybrid approach shows immense potential for improving the processing efficiency and controllability of the microlens morphology at the same time. For example, convex microlens with aspherical morphology and concave microlenses with spherical morphology were realized on glass with good focusing and imaging properties. Therefore, this study presents a new way to fabricate micro-optical components with complex morphology and high surface quality on hard materials.

## Data Availability

The data presented in this study are available on request from the corresponding author.
